# Physicochemical Properties and Digestion of Lotus Seed Starch under High-Pressure Homogenization

**DOI:** 10.3390/nu11020371

**Published:** 2019-02-11

**Authors:** Zebin Guo, Beibei Zhao, Liding Chen, Baodong Zheng

**Affiliations:** 1 College of Food Science, Fujian Agriculture and Forestry University, Fuzhou 350002, China; gzb8607@163.com (Z.G.); nwzj151@163.com (B.Z.); 2 Fujian Provincial Key Laboratory of Quality Science and Processing Technology in Special Starch, Fujian Agriculture and Forestry University, Fuzhou 350002, China; 3 China-Ireland International Cooperation Centre for Food Material Science and Structure Design, Fujian Agriculture and Forestry University, Fuzhou 350002, China; 4 College of life Science, Fujian Agriculture and Forestry University, Fuzhou 350002, China; BioEngin_2015@126.com

**Keywords:** high-pressure homogenization treatment, physicochemical properties, DIVRSD-II model, digestion properties

## Abstract

Lotus seed starch (LS), dispersed (3%, *w*/*v*) in deionized water was homogenized (0–180 MPa) with high-pressure homogenization (HPH) for 15 min. The effects of HPH treatment on the physicochemical properties of the starch system were investigated. The properties were affected by HPH to various extents, depending on the pressure. These influences can be explained by the destruction of the crystalline and amorphous regions of pressurized LS. The short-range order of LS was reduced by HPH and starch structure C-type was transformed into B-type, exhibiting lower transition temperatures and enthalpy. The LS absorbed a great deal of water under HPH and rapidly swelled, resulting in increased swelling power, solubility and size distribution. It then showed “broken porcelain-like” morphology with reduced pasting properties. Digestion of pressurized LS complex investigated by a dynamic in vitro rat stomach–duodenum model showed higher digestion efficiency and the residues exhibited gradual damage in morphology.

## 1. Introduction

Starch is a macromolecule compound composed of glucose. It can be extracted from starchy substances such as corn, lotus seed and sweet potato [1,2,3]. Lotus seed starch (LS) is commercially available in China and it is consumed as a traditional confectionery and food additive. Production of LS is rising year by year because of market demand. However, LS has a poor utilization rate because native starch has a high tendency toward retrogradation and inadequate storage stability at low temperatures [4]. Modified starch, with altered physicochemical properties to improve its functional characteristics, is used to tailor starch to specific food applications [5].

High-pressure homogenization (HPH), in which high pressures are applied over a very short time, involves synchronous application of cavitation, shear, turbulence and temperature rise induced by pressure [6]. Starch was shown to be partly gelatinized after HPH and the degree of gelatinization increased with homogenizing pressure–causing granule size changes [7]. Starches respond differently to HPH according to the pressure applied, starch concentration and source and treatment time. Various effects induced by pressure cause quality changes in starch [6]. However, the application of HPH and functional characteristics of starch have not been commonly studied, particularly with regard to digestion properties.

Research on starch digestion includes in vivo and in vitro methods. The in vivo studies are costly, time-consuming and impeded by ethical issues even though they are relatively accurate ways to study functions of the gastrointestinal tract. In vitro simulation tests used to study digestion have a common flaw in that they oversimplify the gastrointestinal tract structure [8,9]. To solve these problems, a soft-elastic silicone rat stomach model was manufactured using 3D printing technology. An enhanced version of this “near-real” dynamic in vitro rat stomach system, DIVRSD-II (also called Biomimic Rat II, which incorporates the duodenum in a model stomach system) was recently introduced and its digestion of casein and coarse rice grains has proved similar to that of in vivo tests [10,11]. Moreover, the digestion behavior of DIVRSD-II on casein powder suspension is significantly affected by the gastrointestinal tract structure [10].

The published literature–with respect to the digestion properties of starch under HPH–is limited and focuses primarily on digestion rate. A comprehensive investigation addressing the structural changes occurring in starch–water systems before and after digestion is lacking. In this study, the effects of HPH on the physicochemical properties of LS were characterized by scanning electron microscopy (SEM), X-ray diffraction (XRD) measurements, laser diffraction particle size analysis, and Fourier transformed infrared spectroscopy (FTIR). At the same time, the viscosity, thermal properties, solubility and swelling power of homogenized starches were studied. Notably, the DIVRSD-II model was used to study the digestion efficiency of HPH-treated LS and the structural changes both before and after digestion were investigated, which will greatly help in clarifying the relationship between digestion and the structure of LS.

## 2. Materials and Methods

### 2.1. Materials

The LS (Green Field Fujian Food Co. Ltd., Fujian, China) was obtained according to Guo et al. [12], and contained on average (dry weight basis) 10.75% moisture, 0.29% ash, 0.32% protein, and 0.29% lipid.

### 2.2. HPH Treatment

A 3% (*w*/*v*) starch suspension was prepared by adding LS to deionized water at room temperature (27 ± 1 °C). The well-mixed suspension was homogenized in a high-pressure homogenizer (FPG12805L; Stansted Fluid Power, UK) at 0, 60, 90, 120, 135, 150 and 180 MPa for 15 min, respectively. Approximately 500 mL of suspension was processed at each pressure level. The treated samples were freeze-dried (ALPHA 1-4/2-4 LSC plus; Germany christ Ltd., Osterode, Germany) for about 24 h with the condenser temperature at approximately −80 °C, passed through a 100-mesh sieve, then sealed and stored in a desiccator at room temperature.

### 2.3. FTIR Analysis

The FTIR spectra of starch samples were obtained using a Tensor 27 FTIR spectrometer (Bruker, Karlsruhe, Germany). To form a KBr pellet, 2 mg of starch powder and 100 mg of KBr were weighed and crushed in a quartz bowl. The spectra were scanned from 400 to 4000 cm^−1^ with 32 scanning times at resolution 4 cm^−1^.

### 2.4. XRD

Starch structures were determined using a D/max 2200PC X-ray diffractometer (Rigaku Corporation, Tokyo, Japan) at room temperature, with Cu Kα radiation (*k* = 0.154 nm). The patterns were obtained with a scanning range of 5–35° and a scanning speed of 8° min^−1^.

### 2.5. SEM Analysis

A scanning electron microscope (JSM-6360LV, JEOL, Tokyo, Japan) was used to obtain morphology of starch samples, with the starch powder placed on the metal stub using double-sided glue. Morphology was observed under vacuum, with 20 keV accelerating voltage.

### 2.6. SP and Solubility

The starch suspensions (2%, *w*/*v*) were prepared and heated in a boiling water bath with heating temperatures over 55–95 °C to obtain SP and solubility according to Guo et al. [13]. The heated sample was cooled and centrifuged (4000× *g* for 10 min). The precipitated and dried supernatant were weighed respectively. Each group was measured using the following formulae:

(1) $$\mathrm{SP}=\frac{C}{A-B}\times 100\%$$

(2) $$\mathrm{Solubility}=\frac{B}{A}\times 100\%$$

where B is dry weight of the supernatant, A is weight of the starch sample powder and C is mass of the precipitate.

### 2.7. Laser Scattering Measurement

A laser diffraction particle size analyzer (Malvern Mastersizer 3000, Malvern Instruments Ltd., England, UK) was used to assess starch granule size. Distilled water was the dispersant. Starch powder was dispersed in the cavity of the laser analyzer. Once the shading rate reached 8–15%, the particle size was measured three times and averaged.

### 2.8. Rapid Viscosity Analyzer (RVA) Analysis

The 6% (*w*/*v*) starch suspensions were prepared and a RVA (TechMaster, Newport Scientific Pty. Ltd., Australia) used to determine their pasting properties according to Chen, et al., with a little modification [6]. Starch suspensions were equilibrated at 25 °C for 1 min heated to 95 °C at 5 °C min^−1^ and then cooled to 25 °C at the same rate.

### 2.9. Differential Scanning Calorimeter (DSC) Analysis

A 2 mg sample was added to the DSC pan and deionized water was added at a ratio of 1:2 (*w*/*v*). Samples were equilibrated for 24 h at room temperature and then heated using a differential scanning calorimeter (DSC-200FC, NETZSCH, Selb, Germany) from 20 °C to 95 °C at 10 °C min^−1^, with an empty DSC pan used as a reference. The onset, peak, and conclusion temperatures were determined from the DSC gelatinization curve. The gelatinization enthalpy (ΔH) was calculated according to the peak area.

### 2.10. In Vitro Digestion of HPH-Treated Starch in the DIVRSD-II Model

Artificial rat saliva and gastric juice were prepared following the method of Chen et al. [10] and Wu et al. [14]. Artificial pancreatic juice and bile juice were prepared according to Wu et al. [15]. Glucose concentration was determined with a D-glucose assay kit (GOPOD-Format) (Megazyme International Ireland Ltd., Wicklow, Ireland). Gastric mucin and α-amylase were obtained from Sigma (Sigma-Aldrich, Saint Louis, MO, USA). Amyloglucosidase, pancreatin, and pepsin were obtained from Solarbio (Shanghai Solarbio Bioscience & Technology Co. Ltd., Shanghai, China). All other chemical reagents were obtained from Sinopharm Chemical Reagent Co. Ltd. (Beijing, China).

The DIVRSD-II model was applied to digestion of HPH-treated starch [15]. Starch samples (200 mg, dry basis) were dispersed in 2.0 mL of water and cooked for 15 min until gelatinized. Then 2.0 mL of artificial saliva (37 °C) was added to mimic oral digestion [16]. The food samples were digested in the DIVRSD-II model in batches as reported by Wu et al. [15] for 0, 10, 20, 30, 40, 50, 60, 90, 120 and 180 min.

### 2.11. Statistical Analyses

Experimental diagrams were produced with Origin Pro 8.5. Data processing and significant difference analysis were performed with DPS 9.05 191 (Science Press, Beijing, China). Differences among experimental mean values (*p* < 0.05) were determined by Duncan’s multiple range test.

## 3. Results and Discussion

### 3.1. XRD Patterns

**Figure 1:**
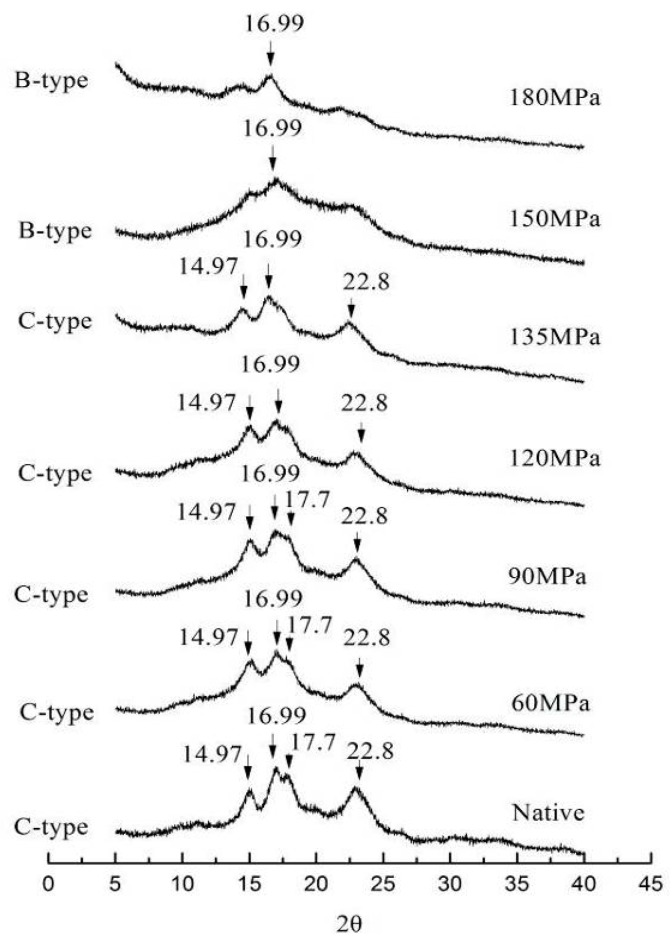
X-ray powder diffraction patterns of native and pressurized lotus seed starch.

The XRD patterns of LS subjected to HPH are shown in Figure 1. Native LS had strong diffraction peaks at 2θ values (14.97°, 16.99°, 17.70° and 22.80°), indicating a C-type starch [13]. There were no marked changes in XRD patterns of starch granules for 60 MPa HPH. However, as pressure increased, the intensity of diffraction peaks at 14.97°, 17.70° and 22.80° decreased; the peak at 17.7° disappeared when pressure attained 135 MPa, which contributed to a loss in crystallinity [17]. Following HPH treatment at 150 and 180 MPa, all XRD peaks became very weak except for a strong peak at 16.99°, suggesting a B-type starch. Similar results were reported concerning the structure of pressurized maize starch (A-type) being transformed into B-type patterns [18]. The HPH not only affects the amorphous zone of starch granules, but also destroys its crystalline zone. This is probably due to cavitation induced by HPH–cavities filled with gas or vapor take shape as pressure decreases and collapse immediately when the pressure increases once more. High pressure gradients and high local velocities of liquid layers are induced in the vicinity of cavitation bubbles with their sudden collapse, which destroys the original structure of starch [19,20]. Additionally, cavitation decomposes water into OH radicals and H atoms and induces further interaction between radicals and starch molecules, thus transforming their structure [7].

### 3.2. FTIR Analysis

**Figure 2:**
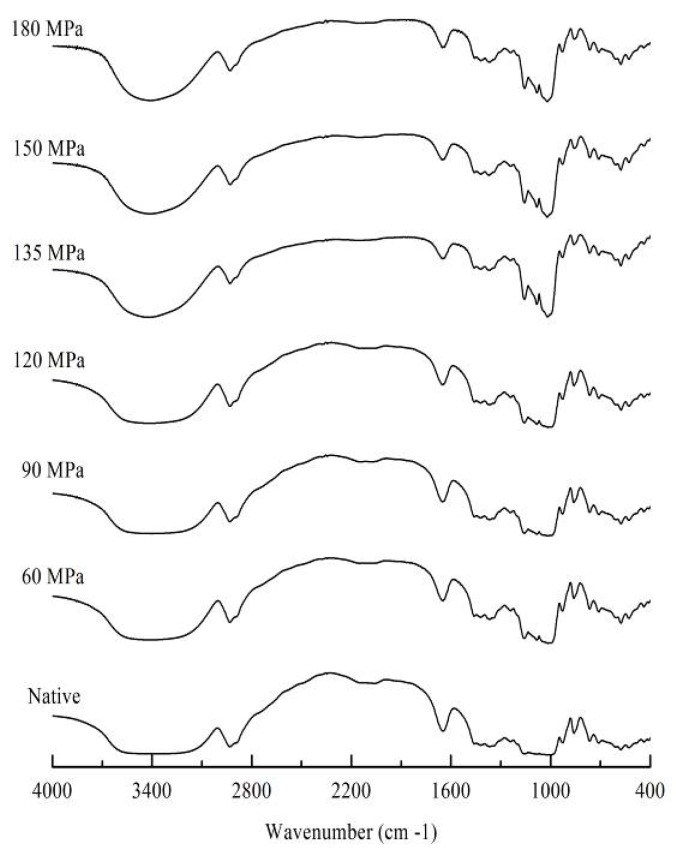
Fourier transformed infrared spectroscopy (FTIR) of native and pressurized LS.

The FTIR was used to detect changes in starch structure on the molecular level. The FTIR spectrum changes can be divided into changes in band narrowing and in band intensity and relate to the ordering of specific conformations, such as the IR bands at 1022 cm^−1^—that are thought to be related to short-range order of starch [21]. In the current investigation, the absorbance ratio of 1022/995 cm^−1^ was used as an index of structure order. The higher is the molecular order of starch double-helices structure, the lower is the absorbance ratio of 1022/995 cm^−1^ [22]. The absorbance ratio of 1022/995 cm^−1^ increased as pressure was elevated (Table 1). This suggested that HPH destroyed starch structure and reduced their short-range order. Meanwhile, pressure-dependent band narrowing emerged at around 3400 cm^−1^ (Figure 2), further indicating that starch structure was seriously affected by HPH treatment. These results were in accordance with the XRD results.

### 3.3. Morphology

**Figure 3:**
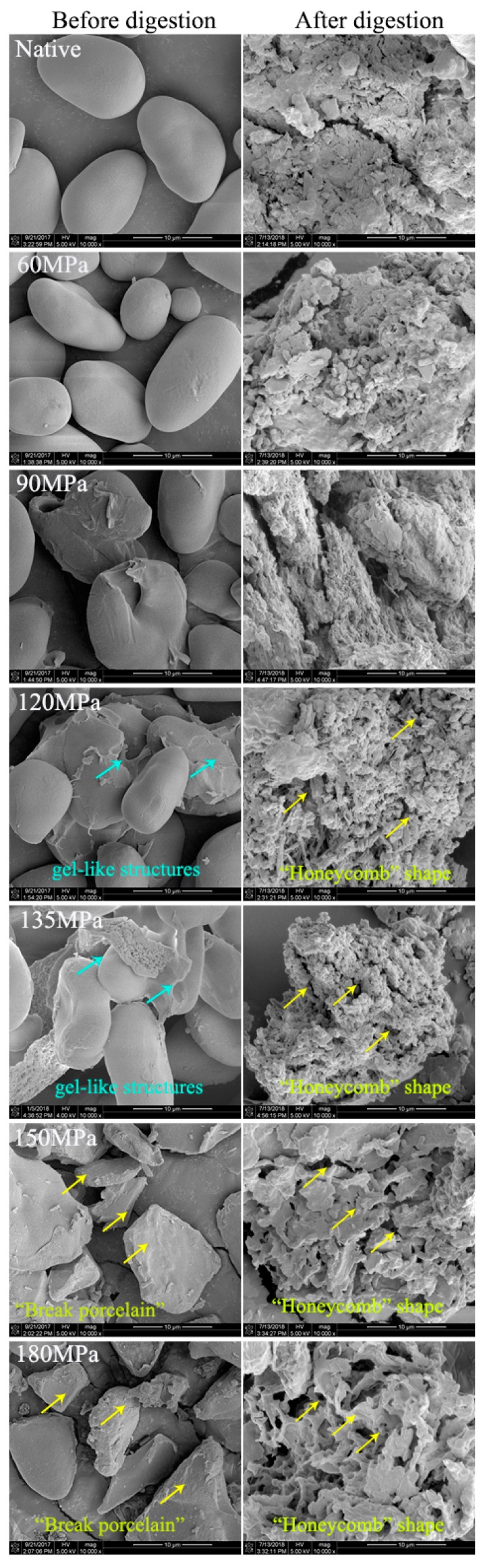
Scanning electron microscopy (SEM) of native and pressurized LS before digestion (10,000×) and after digestion (10,000×) in the DIVRSD (dynamic in vitro rat stomach system) model for 180 min.

There was an evident change in the starch morphology when subjected to HPH. Most of the native starch granules had an elliptical shape with a smooth surface and most retained their granule structure in HPH-treated starch up to 60 MPa (Figure 3). However, during treatment at 90 MPa, some ruptured starch granules had slightly swollen surfaces and aggregated with other granules, forming gel-like structures; this became more pronounced when pressure reached 120 MPa, indicating that granule structure was destroyed by the HPH. At 135 MPa of treatment there were obvious changes in diffraction peaks (Figure 1, 135 MPa), and LS showed significant deformation and partial gelatinization, as the HPH linearly increased the water temperature [23,24]. A large proportion of granules may have contained two different regions: the outer region remaining unchanged while the internal region was significantly altered. When pressure exceeded 150 MPa, all granules lost their normal granule structure and broke into fragments, showing “broken porcelain-like” structures. In addition to the cavitation mentioned above, there were two other reasons for granule degradation induced by HPH: (1) shear forces induced by HPH can interrupt covalent bonds that bind the starch chains, as long as the chains are beyond a critical value, in what is known as mechano-chemical action of HPH; and (2) changes of pressure result in rapid, instantaneous temperature increases in the thin liquid layers adjoining cavities, thus polymers are unavoidably pyrolyzed [19]. Not only cavitation, but shear and turbulence induced by high pressure also increase as homogenizing pressure rises, which makes breaking of polymers much easier [25,26,27]. This suggests that HPH is a good method for modifting starch.

### 3.4. SP of Starch Granules

**Figure 4:**
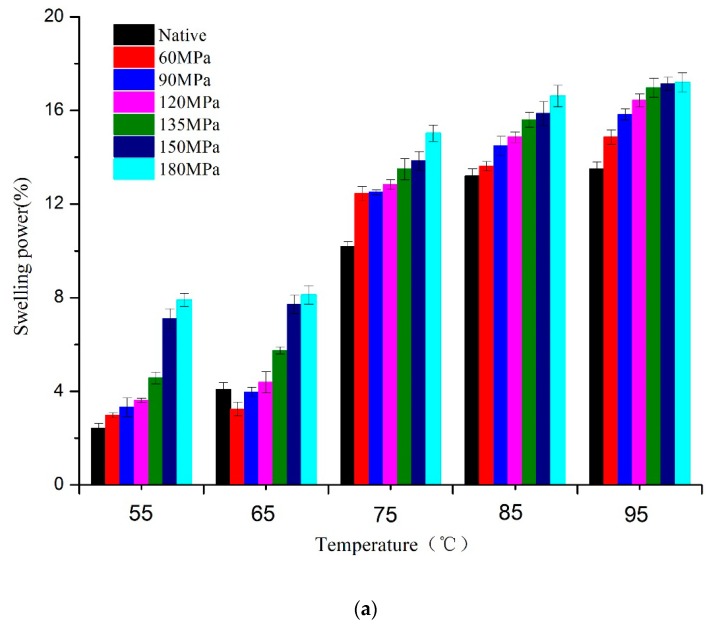

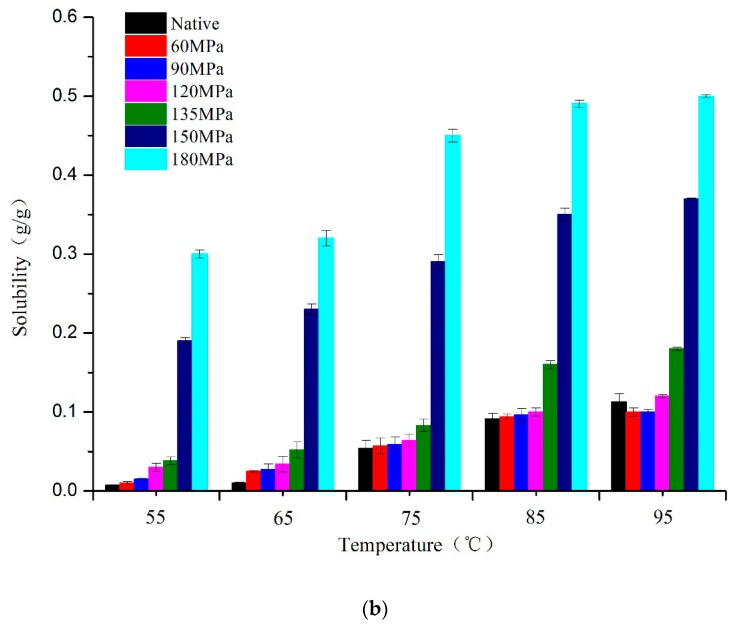
Swelling power (**a**) and solubility (**b**) of native and pressurized LS.

The SP and solubility provide information for assessing the degree of interaction between starch chains within the amorphous and crystalline domains of the starch granule. There was a significant increase in SP of all samples at 75 °C (Figure 4), indicating that starch molecules combined with water molecules activated by heat and gelatinization occurred. Obviously, the SP of HPH-treated samples would gradually increase with rising pressure at any temperature stage, suggesting that HPH can advance water transfer into starch granules and therefore further influence the swelling of amorphous regions. This is consistent with previous studies [17,23], showing that starch granules being homogenized can absorb a great deal of water and swell rapidly. Because of the higher SP described above, starch granules were more vulnerable to shear stress induced by HPH, with some amylose leaching out even though starch retained granular structures and thus showed greater solubility than native starch (Figure 4a). The amount of soluble carbohydrate increased markedly at 75 °C due to the irreversible rupturing of starch granules. The ruptured fragments tended to readily form aggregates during heat-induced gelatinization. Treatment with HPH has been shown to dissociate solution aggregates [28]. Some amylopectin may be decomposed into amylose by HPH, which also contributes to the pressure-dependent increasing of solubility (Figure 4b).

The average size distribution of starch particles under different HPH conditions is shown in Table 1. D(3,2) (area mean diameter.)represents the area mean diameter. The starch granule size increased as pressure was elevated. This was the result of the high pressure and instantaneous temperature rise mentioned in relation to SEM and, under their influence, starch granules can absorb a great deal of water and rapidly swell, causing the size increase. Similar results were previously reported [17,23], in which pressurized starch showed a pronounced size increase compared to native cassava starch. Although the increased shear force induced by high pressure produced some smaller pieces in our study, there was only a non-significant decrease in particle size. This is consistent with the SEM results.

### 3.5. Pasting Properties

**Figure 5:**
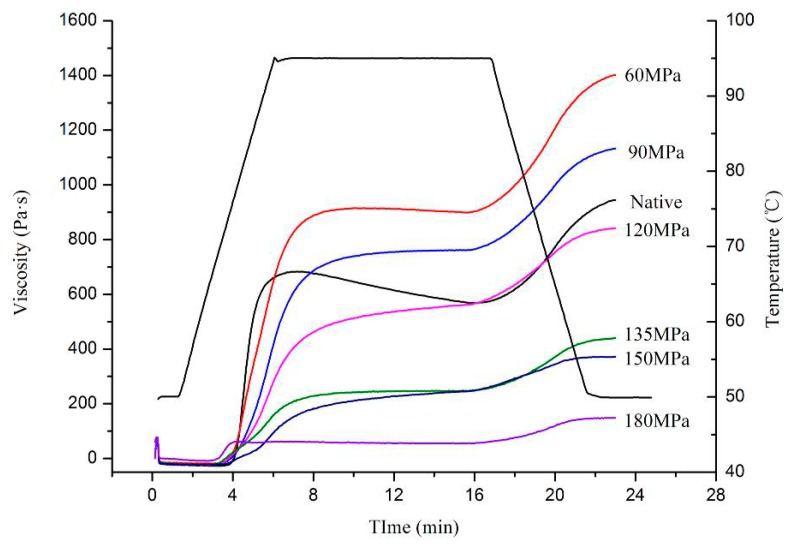
Pasting properties of native and pressurized LS.

The 6% (*w*/*v*) homogenized-starch suspensions were determined by RVA and their pasting properties are summarized in Figure 5. The peak viscosity (PV), trough viscosity (TV), and final viscosity (FV) values of HPH-treated (120–180 MPa) starch gradually decreased as the pressure increased, except for 60 and 90 MPa. At pressures above 120 MPa, peaktime (PT) was not detected, suggesting destruction of the crystalline region inside the starch. This may be due to starch granule changes during the re-formation of crystalline structures [13,29]. The increasing temperature and cavitation induced by HPH hastened the movement of molecules, and so accelerated reactions between starch molecules, therefore starch gelatinization occurs However, cavitation, shear and turbulence phenomena became even larger as homogenizing pressure increased, which destroyed intermolecular bonding between starch molecules and so could interrupt covalent bonds that bound the chains of starch, especially double-helical structures of amylopectin crystallites, as long as the chains exceeded a critical value [30,31]. The water molecules also acted as diluents, not only interrupting aggregation of starch molecules of low viscosity, but providing a chance for granules to react with them. This finally transformed the native C-type into a B-type pattern as described for the XRD.

### 3.6. Thermal Properties

The thermal properties of native and HPH-treated LS were measured using a DSC (Table 1). There was a considerable reduction in the values (To, Tp, Tc, and ΔH) of HPH-treated (60–180 MPa) starch compared to native LS, indicating that granule hydration occurred with HPH treatment. This was in accordance with the SEM results. The ΔH represents the thermal energy needed to melt double-helical structures of amylopectin crystallites [32]. The HPH treatment advanced the double-helical dissociation, weakening the molecular order and crystalline structure. In contrast, native starch samples need higher energy to destroy intermolecular bonding between water and starch molecules, such as hydrogen bonds and starch crystallites [33]. Additionally, HPH treatment of >150 MPa changed the XRD of starch to a B-type pattern (Figure 1). The B-type starches tend to exhibit lower transition temperatures and enthalpy [34,35]. The DSC investigation further indicated that HPH may be a superior method for starch modification.

### 3.7. Starch Digestion by DIVRSD-II

**Figure 6:**
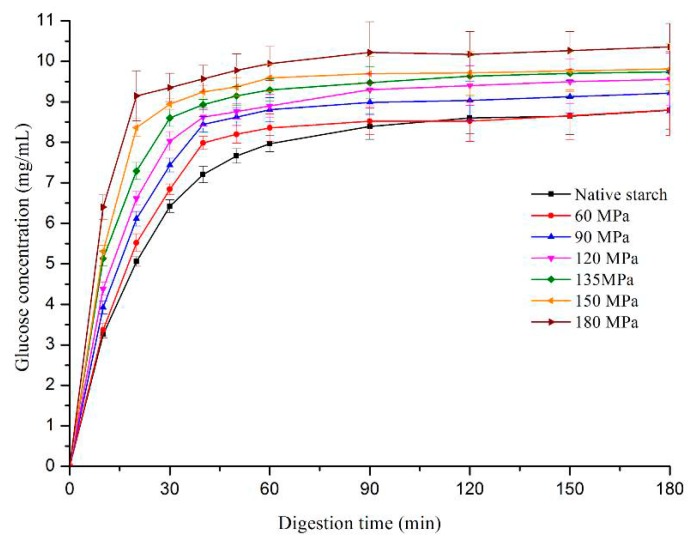
Digestion efficiency of native and pressurized LS.

The digestion efficiency of HPH-treated starch was compared with native LS. The enzyme susceptibility of HPH-treated starch was higher than that of native LS and was in the following order: 180 > 150 > 135 > 120 > 90 > 60 MPa > native starch (Figure 6). Digestion efficiency of starch relies on various factors, such as size and damage degree of granule, crystallinity and proportions of acmylose and amylopectin [36,37]. Native starch had a much higher degree of crystallinity, as well as double-helical order and so its digestibility was lower than HPH-treated starch, even if the HPH treatment had somewhat increased the starch granule size. Also, amylose molecules tend to be amorphous in starch granules and high shear forces of HPH were shown to disrupt the integrity of starch granules and decompose some amylopectin into amylose [26]. These were all reasons for the higher digestion efficiency of HPH-treated starch.

Great variation between native and HPH-treated starch was observed in SEM micrographs (Figure 3). After 180 min of digestion, a large amount of small detritus appeared on the surface of dense particles of native starch, indicating enzymolysis. There were no significant changes in starch with moderate HPH treatment (60 MPa) compared to native starch, consistent with the digestion efficiency. However, for pressure >90 MPa, the small detritus was replaced by holes of varied sizes on the surface and in the interior of granules, showing an irregular “honeycomb” shape and loose organizational structure—with greater pressure, the voids were larger. This was due to severe damage to the crystalline and amorphous regions of starch with HPH, leaving both inside and outside of the fragments more vulnerable to amylase and resulting in higher digestion efficiency compared with native starch.

Due to the high efficiency and the dramatically greater yield of modified starch, we believe that HPH has a great potential for academic and industrial research activity. It is an effective technique for modified starch preparation and a new strategy for process intensification, enhancing competition of industries to be more economic and innovative.

## 4. Conclusions

In this study, the effects of HPH treatment (up to 180 MPa) on the physicochemical and digestion properties of LS were investigated. Cavitation, shear and turbulence induced by high pressure caused irreversible distortion to crystalline and amorphous regions of LS, thus transforming the C-type into B-type starch structure and exhibiting lower transition temperatures and enthalpy. The absorbance ratio of 1022/995 cm^−1^, as an index of structure order, showed a pressure-dependent increase for FTIR, indicating severe damage of pressurized LS short-range order. The SEM showed evident change in starch morphology, with aggregation of ruptured starch granules, forming gel-like structures following >90 MPa HPH. However, at >150 MPa, granules broke into fragments, showing “broken porcelain-like” structures. The LS absorbed a great deal of water during HPH treatment and rapidly swelled, thereby increasing size distribution of pressurized LS and showing increased SP and solubility. The RVA analysis showed that most pressurized LS had a lower viscosity than native starch, because starch chains, especially double-helical structures of amylopectin crystallites were broken by cavitation, shear and turbulence phenomena. The severe damage to starch granules as well as the lower degree of double-helical order resulted in higher digestion efficiency of LS under the DIVRSD-II model. Additionally, both the inside and outside of starch fragments were vulnerable to amylase and the residues exhibited an irregular “honeycomb” shape in morphology and the amount of residual starch decreased with increased pressure.

## Figures and Tables

**Table 1:** Thermal properties, particle size and Fourier transformed infrared spectroscopy (FTIR) ratio of Native and pressurized LS.

Pressure	1022/995 cm^−1^	D(3,2)	To	Tp	Tc	ΔH
Native	0.85 ± 0.06 ^b^	11.50 ± 1.80 ^e^	69.01 ± 0.11 ^a^	72.68 ± 0.21 ^a^	83.00 ± 0.34 ^a^	13.28 ± 0.41 ^a^
60	0.97 ± 0.05 ^a,b^	12.00 ± 1.50 ^d,e^	68.43 ± 0.30 ^a^	71.99 ± 0.21 ^b^	82.45 ± 0.44 ^a^	9.94 ± 0.35 ^b^
90	0.97 ± 0.09 ^a^	12.10 ± 1.84 ^d,e^	66.01 ± 0.24 ^b^	71.49 ± 0.07 ^c^	78.66 ± 0.25 ^b^	8.89 ± 0.50 ^c^
120	0.98 ± 0.07 ^a^	16.20 ± 2.10 ^c,d^	65.70 ± 0.21 ^b,c^	70.74 ± 0.16 ^d^	76.35 ± 0.42 ^c^	7.07 ± 1.27 ^d^
135	1.03 ± 0.06 ^a^	18.10 ± 3.20 ^b,c^	65.22 ± 0.49 ^c^	70.49 ± 0.28 ^d^	75.64 ± 0.27 ^c^	5.33 ± 0.10 ^e^
150	1.00 ± 0.04 ^a^	22.70 ± 2.70 ^a,b^	51.82 ± 0.61 ^d^	56.59 ± 0.41 ^e^	59.92 ± 1.00 ^d^	5.18 ± 0.12 ^e^
180	1.00 ± 0.05 ^a^	21.00 ± 2.65 ^a^	49.41 ± 0.36 ^e^	56.22 ± 0.09 ^e^	56.75 ± 0.44 ^e^	5.08 ± 0.18 ^e^

Experimental data are expressed as mean ± SD (*n* = 3). Different letter following the values within the same column present significant differences (*p* < 0.05).
